# Objectively Measured Physical Activity and Sedentary Time among Children and Adolescents in Morocco: A Cross-Sectional Study

**DOI:** 10.1155/2018/8949757

**Published:** 2018-09-26

**Authors:** Issad Baddou, Asmaa El Hamdouchi, Imane El Harchaoui, Kaoutar Benjeddou, Naima Saeid, Mohammed Elmzibri, Khalid El Kari, Hassan Aguenaou

**Affiliations:** Unité Mixte de Recherche Nutrition et Alimentation CNESTEN-Ibn Tofail University (URAC39), Regional Designated Center of Nutrition Associated with AFRA/IAEA, Morocco

## Abstract

**Background:**

Regular physical activity in childhood and adolescent plays an important role in reducing the risk of cardiovascular health diseases, diabetes, and obesity in adulthood. However, little is known about physical activity levels (PA) and sedentary time among children and adolescents in Morocco.

**Objective:**

To examine gender, type of day, and age grade differences in objectively measured sedentary time, physical activity levels, and physical activity guideline attainment among children and adolescents in Morocco.

**Method:**

172 children/adolescents (mean age = 10.92 ± 1.55 years, 49.4% are boys) were recruited for this study and wore a tri-axial accelerometer (GT3X+) for 7 consecutive days. Time spent in sedentary, PA levels, and daily steps were measured and compared according to gender, age grade, and the type of day (weekdays/weekends).

**Results:**

In weekdays children/adolescents spent more time in sedentary than weekends (p < 0.001). Boys were eight times more likely to meet the recommendation for at least 60 min of moderate to vigorous physical activity per day than girls (OR: 8.569; 95% [CI]: 4.23–17.32), p < 0.001.

**Conclusion:**

These findings highlight the need for effective and sustainable strategies and programs aiming to promote physical activity and to reduce sedentary behavior among children and adolescents in Morocco.

## 1. Introduction

Physical inactivity is the fourth leading cause of death in the world [1]. Lack of physical activity is an important contributing factor in the development of childhood obesity [2]. Overweight children and adolescents are more likely to become obese and to develop noncommunicable diseases (NCDs) during adulthood [2]. A total of ≥60 min of moderate to vigorous physical activity (MVPA) each day is recommended for children aged 6 to 17 years [3]. Reduction in physical inactivity would reduce between 6% and 10% of the major NCDs and increase life expectancy [4].

In high-income countries (HIC), surveillance of children and adolescents physical activities using objective method has been taken for decades [5]. In contrary to Africa, few studies have used objective methods to assess PA among children and estimates of physical activity or sedentary behavior has derived mostly from self-report questionnaires. Indeed, a review of Peltzer reported that only 14.2% of children from 8 countries were physically active (5 days or more in a week with at least 60 min/day) and there are large differences among countries meeting the PA guidelines, 17.7% in Uganda and 9.0% in Zambia through self-report questionnaires [6], 3.9% in Mozambique [7], 1% in South Africa using GT3X accelerometer [8], and 54.8% in Senegal using GT3X+ accelerometer [9].

In Morocco, physical activity in children and adolescents has been measured only with self-report questionnaires [10, 11]. However, activity information derived from self-report is potentially subject to response bias [12] and thus accurate assessment is required to assess current and changing physical activity levels.

As known, physical activity is complex behavior that tends to vary considerably from children or adolescents to another according to different factors. Previous studies assessing objectively measured physical activity among children and adolescents suggest that boys are more active than girls [13, 14] and physical activity levels decline with age, especially during late primary school years [14]. In Morocco, this study is the first of its kind to use GT3X+ accelerometer to assess physical activity and sedentary time among children and to examine gender, age grade, and type of day differences in physical activity level, sedentary time, and attainment of recommended physical activity guidelines among children and adolescents.

## 2. Methods 

### 2.1. Ethics Statement

The study was approved by the Ethical Committee of Biomedical Research (CERB). Written informed consent was obtained from the parents of the participants.

### 2.2. Participants

Participants were a convenience of 175 children/adolescents, who were recruited from urban schools from three cities of Morocco. The study was carried out during school-week, and accelerometry data were collected over seven consecutive days.

To obtain information on children's age, sex, home address, nationality, parental work, and household size, a short parental questionnaire was handed out to assess factors of children's individual socioeconomic status.

### 2.3. Anthropometry

Anthropometrics measurements were performed by trained operators according to international recommendations [15]. Weight was measured to the nearest 0.1 kg using an electronic portable scale (SECA “Seca Weighing and Measuring Systems, Hamburg”, Germany) with the children/adolescents barefoot and wearing light clothes. Height was measured to the nearest 0.1 cm using a stadiometer (ShorrBoard, Portable Height-Length Measuring Board) with the children/adolescents barefoot in a standing position.

### 2.4. Assessment of Physical Activity

Physical activity was measured using the Actigraph GT3X+ (Pensacola, Florida, USA) a triaxial accelerometer that captures acceleration movements in three axes (Vertical, Horizontal, and Perpendicular) [16].

Children/adolescents were instructed to wear the accelerometer attached to an elasticized belt around the waist, positioned just above the right hip, once they woke up until bed time at night for 7 consecutive days and to remove the accelerometer any time they were to perform activities that involve the use of water such as bathing or swimming, and when going to bed. The accelerometer was initialized to store activity counts every 15 seconds (i.e., for 15-second epochs).

Children/adolescents had to have at least 4 valid days of data (3 week days and 1 weekend day). A valid day is a day with ≥ 600 min of registered data. The cut point used to determine the level of physical activity is the cut point developed by Evenson [17]. The levels of PA were expressed in count per minute (cpm): sedentary (0–100 cpm), light (101–2295 cpm), moderate (2296–4011 cpm), and vigorous (≥4012 cpm). The Actigraph filter was used to separate physical activity level according to the type of day (weekends, weekdays).

The Actigraph data were downloaded using the software provided by the manufacturer (version 6.13.2, Actilife).

### 2.5. Statistical Analysis

The Kolmogorov Smirnov test was performed to check the distribution of the variables; all variables were normally distributed and were expressed as the mean with standard deviations. t-test was used to examine the difference between weekends and weekdays.

In order to analyze the difference in physical activity levels and sedentary time by age grade, we divided the participants into two subgroups: children [8-11[ years and adolescents [11-14] years. Two-way analysis of covariance was used to examine gender and age grade differences in physical activity level and sedentary time variables separately for weekdays and weekends, adjusted for body mass index for age (BMI z-score) and wear time. Effect sizes (*η*2) were also calculated to examine the practical significance of these differences. Logistic regression analyses were conducted to examine independent relationships between attainments of physical activity guidelines and both gender and age grade. All statistical analyses were performed using Statistical Package for the Social Sciences (SPSS, version 21) and results were considered significant at p < 0.05.

## 3. Results

Among the 175 children/adolescents recruited, 3 children/adolescents were excluded from the analysis because of invalid day.

### 3.1. Description of Children/Adolescents Characteristics

General characteristics of children/adolescents are shown in Table 1.

Children/adolescents were aged between 8 to 14 years (mean age = 10.92 ± 1.55 years). 49.4% were boys. 50 % were aged between 8 and 11 years.

The mean BMI z-score was -0.16 ± 1.33. There were no significant differences in children/adolescents characteristics according to gender.

### 3.2. Description of Physical Activity Levels and Sedentary Time according to the Type of Day

Physical activity levels of children/adolescents according to the type of day are shown in Table 2.

On weekdays, children/adolescents had worn the accelerometer for an average 871.22 ± 75.33 (min/day), with 559.76 ± 90.75 (min/day) of the time in sedentary and average of 55.25 ± 26.30 (min/day) in MVPA (Table 2).

On weekends, children/adolescents had worn the accelerometer for an average 797.64 ± 109.12 (min/day), with an average of 59.92 ± 33.76 (min/day) in MVPA (Table 2).

Children/adolescents spent more time in sedentary during weekdays than weekends (p < 0.001). However no differences were seen between weekdays and weekends in MVPA.

During weekdays, children/adolescents accumulate 10484.82 ± 3554.86 steps per day, while in weekends they accumulate 10742.19 ± 4436.79 of steps per day.

Children/adolescents accumulate less count per minute in weekdays than weekends (p < 0.001).

**Figure 1:**
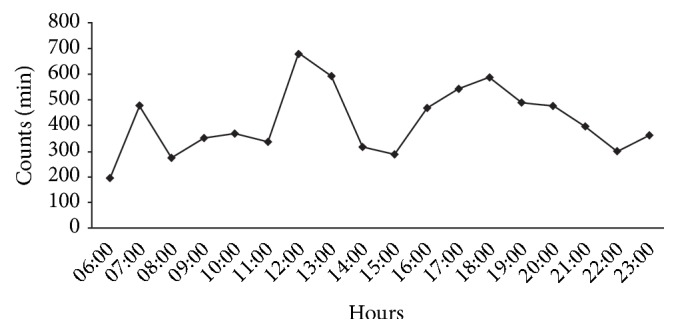
Cumulative mean physical activity counts per minute over the weekdays.

Activity data (count/min) in weekdays are depicted in Figure 1. The activity curve (count/min) is illustrative of the typical activity pattern in everyday life of children examined during only weekdays, with characteristic peaks and troughs throughout the day. Activity seems on average lower during school time (8h00 to 12h00 and 14h00 to 16h30) than after school.

Figure 1 represents the average accelerometry count/minute of children and adolescent during weekdays.

### 3.3. Differences in Physical Activity Levels and Sedentary Time by Gender and Age Grade on Weekdays and Weekends

Table 3 shows the differences in physical activity levels and sedentary time by gender and by age grade on weekdays.

On weekdays, when differences in PA levels and sedentary time were analyzed for gender we observed that boys were more engaged in moderate (p < 0.001) and vigorous (p < 0.001) activity and took more steps (p < 0.001) than girls whereas girls were more engaged in sedentary time than boys (p = 0.004).

When PA levels and sedentary time were analyzed for age grade we observed that adolescents spent more time in sedentary (p = 0.001) and less time in light activity (p < 0.001) than children, whereas no difference according to age grade was observed in MVPA (p = 0.255).

Small effect of size of gender was observed in vigorous activity (*η*2 = 0.213).

Table 4 shows the differences in physical activity levels and sedentary time by gender and by age grade on weekends.

During weekends, when differences in PA levels and sedentary time were analyzed for gender we observed that boys were more engaged in moderate (p < 0.001) and vigorous (p < 0.001) activity and took more steps (p = 0.001).

When PA levels and sedentary time were analyzed for age grade, we observed that adolescents spent more time in sedentary (p = 0.001) and less time in light activity (p < 0.001) than children, whereas no difference according to age grade was observed in MVPA (p=0.258).

There are no interactions between gender and age grade with sedentary time (*η*2 = 0.001, p = 0.669 on weekdays; *η*2 = 0.001, p = 0.760 on weekends) and also with all physical activity levels, for example, with light activity time (*η*2 = 0.006, p = 0.332 on weekdays; *η*2 = 0.002, p = 0.571 on weekends) and also with steps (*η*2 = 0.008, p = 0.257 on weekdays; *η*2 < 0.001, p = 0.899 on weekends).

### 3.4. Attainment of Moderate to Vigorous Physical Activity Guideline by Gender and Age Grade

Table 5 shows the odd ratio of moderate to vigorous physical activity guideline attainment.

38.8 % of the participant attained the recommended MVPA guidelines (≥ 60 min/day) where boys were 8 times more likely to meet this recommendation than girls (OR: 8.569; 95% confidence interval [CI]: 4.23–17.32).

## 4. Discussion

This is the first study in Morocco to objectively assess sedentary time and physical activity levels according to gender, age grade, and type of day in children and adolescents with GT3X+ accelerometer. The results of this study suggest that children and adolescents spent more time in sedentary during weekdays versus weekends. The amount of time spent in moderate to vigorous activity is higher among boys than girls.

Several studies [13, 18–21] have examined the time spent in different intensities using objective measures of activity. In the present study, the proportion of time spent in sedentary constituted the majority of the day in these children/adolescents. This makes sense because children/adolescents were sitting in class much of the school days.

Comparisons across the studies for weekdays versus weekends are somewhat difficult because the subject samples and measurement tools differ, as well as the expression of the activity data. Studies have reported differences in activity on weekdays and weekends using objective methods in children and adolescents [18, 19, 22].

Our results revealed that children and adolescents spent more time in sedentary in weekdays versus weekends. Similar trends were reported in different studies [18, 19]. Indeed, a study conducted in four European countries (Denmark, Portugal, Estonia, and Norway) using MTI accelerometer found that time spent in sedentary were higher during weekdays compared with weekends (p < 0.01) in a sample of children and adolescents aged between 9 and 15 years [18]. Likewise, another study conducted in China using GT3X+ or GT3X accelerometer reported that children and youth (mean age = 13.4 years) had more sedentary time on weekdays than on weekend days (p < 0.05) [19].

Sedentary activity in one study [22] as measured by multisensory accelerometer (Actiheart) reported higher means on weekends than on weekdays in children aged 7.1 years.

The relatively lower amount of time spent in MVPA was also documented [19, 23, 24]. A study [23] comparing MVPA in weekdays versus weekends between Liverpool and Madrid youth (aged between 10 and 14 years) using GT1M accelerometer reported that youth in Liverpool spent an average of 48.6 ± 1.5 (min/day) in MVPA, while Madrid youth accumulated an average of 50.6 ± 1.5 (min/day) of MVPA in weekdays. Time spent in MVPA in weekends was 36.5 ± 1.7 (min/day) in Liverpool's youth and 32.3 ± 1.7 (min/day) in Madrid's youth. This would be comparable to our children/adolescents, where the MVPA during the weekdays averaged 55.2 ± 26.3 (min/day) and during weekends, MVPA averaged 59.9 ± 33.7 (min/day), suggesting that children/adolescents in our study were more active.

In the present study, differences in MVPA between weekdays versus weekends were not observed in 8- to 14-year-old children/adolescents while it showed a difference between boys and girls for PA engagement. These findings are similar to results of some studies, which showed that girls are less active than boys [19–21, 25]. For example, Trost et al. examined age- and sex-related PA using accelerometer in children from grades 1-12. They observed that boys are more physically active than girls at moderate and vigorous intensity. In the present study, boys were more likely to spend time on moderate and vigorous physical activities and less time on sedentary than girls.

Our results also revealed that boys took more steps per day and were more likely to meet the recommendation for at least 60 min of moderate to vigorous physical activity per day than girls, in agreement with previous studies [13, 22, 26, 27]. The differences noted may be due to the weakest participation of girls in organized sport [28]. Biological reasons may also contribute to these differences [29].

In addition, our results indicated that sedentary time increases significantly with age grade while light physical activity decreases significantly with age grade, on both weekdays and weekends.

However, on both weekdays and weekends, no differences were observed in MVPA (min/day) according to age grade. These findings are in contrary to results of some studies, which show that MVPA decreases with age [30, 31]; this could be explained by the narrow age range used in this study.

The WHO [3] recommends that at least 60 minutes of daily accumulated MVPA is necessary for the maintenance of metabolic health [3]. Data in the present study showed that neither children nor adolescents satisfied the criterion of accumulating at least 60 minutes of at least moderate intensity PA. Indeed, only 38.8% of the children and adolescents met the recommendation of ≥ 60 (min/day) of MVPA. It appears that PA of primary school participants on the weekdays was well below the recommended 60 minutes.

In conclusion, in both second- and sixth-grade participants we could find several differences in the PA levels between weekdays and weekend days. This study revealed that Moroccan children and adolescents spent more time in sedentary in weekdays than weekends. Boys engaged more in moderate and vigorous intensity levels than girls. These differences emphasize the need of reducing sedentary during school time for future interventions in Morocco, especially when children grow older and for girls. Further studies need to confirm whether the present findings also apply in rural and suburban populations and in different seasons of the year.

### 4.1. Strengths and Limitations

This is the first study in Morocco which objectively assessed PA levels by means of accelerometry (GT3X+) in school children/adolescents and investigated whether there are differences between weekdays and weekend days in two different age groups and according to gender.

However, the present study has several limitations. We assessed the PA levels of a convenience sample of 2nd grade to the 6th grade from three urban regions and surveyed them during school year (winter, spring months), respectively. Therefore, findings are not generalizable to other populations and seasons.

The sample size was relatively small but constitutes the baseline data which are the basis for implementing future monitoring studies with GT3X+ accelerometer in a large population-based surveillance system of youth.

Accelerometry is a good method to gain further insights in children's PA levels. However, the use of accelerometry is associated with several issues: its inability to assess accurately water activities and activities in which the body's centre of gravity is relatively fixed (upper movements) such as cycling [32]. The absence of a universal consensus on data cleaning and processing and accelerometer cut points for determining MVPA [32]. The inclusion criterion of at least 4 valid weekdays and 1 weekend day of 10 valid hours of combined data was chosen to maximize data retention for analysis and enhance the reliability of the presented results.

## Figures and Tables

**Table 1:** General characteristics of the children/adolescents participants in the study presented by gender (mean ± SD).

	**Overall **	**Boys **	**Girls **	**p value**
	**(n=172)**	**(n=85)**	**(n=87)**	
**Age **(years)	10.92 ± 1.55	10.89 ± 1.50	10.96 ± 1.61	0.793
**Weight **(kg)	35.00 ± 9.51	34.05 ± 7.06	35.93 ± 11.37	0.195
**BMI z-score **	-0.16 ± 1.33	-0.18 ± 1.33	-0.14 ± 1.34	0.834

Values are mean ± SD, p values relate to gender difference, and p values derived using t-test.

**Table 2:** Physical activity levels and sedentary time according to the type of day.

	**Weekdays**	**Weekends**	**p value**
**Sedentary time **(min/day)	559.76 ± 90.75	471.35 ± 127.73	< 0.001
**Light intensity physical activity **(min/day)	256.20 ± 52.68	266.36 ± 64.70	0.111
**Moderate physical activity **(min/day)	39.95 ± 18.01	43.26 ± 23.65	0.145
**Vigorous physical activity **(min/day)	15.30 ± 10.67	16.65 ± 13.02	0.292
**MVPA **(min/day)	55.25 ± 26.30	59.92 ± 33.76	0.154
**Steps per day**	10484.82 ± 3554.86	10742.19 ± 4436.79	0.553
**Counts **(Counts/min)	461.67 ± 159.55	557.11 ± 224.47	< 0.001

Values are mean ± SD, p values relate to the type of day difference, and p values derived using t-test.

**Table 3:** Physical activity levels and sedentary time by gender and by age grade on weekdays.

			**Sedentary time**		**Light activity**		**Moderate activity**		**Vigorous activity**		**Steps**	
			(min/day)		(min/day)		(min/day)		(min/day)		(steps/day)	
			Boys	Girls	Boys	Girls	Boys	Girls	Boys	Girls	Boys	Girls
**Children**	Mean		526.4	559.3	277.1	264.7	48.8	35.7	18.8	11.3	11514.4	10005.1
	95% CI	Lower	506.7	540.6	261.6	249.9	43.7	30.8	15.9	8.5	10469.8	9008.03
		Upper	546.1	578.1	292.5	279.4	53.9	40.6	21.7	14.1	12559.1	11002.1
**Adolescents**	Mean		563.8	588.4	240.5	243.1	45.3	30.1	21.4	9.5	11558.3	8868.9
	95% CI	Lower	544.7	568.9	225.5	227.8	40.3	25.1	18.6	6.6	10547.5	7838.2
		Upper	582.8	607.8	255.5	258.3	50.2	35.1	24.3	12.4	12569.1	9899.7
	Gender	F	8.77		0.41		31.5		44.9		16.6	
		p-value	0.004		0.519		< 0.001		< 0.001		< 0.001	
		*η*2	0.050		0.003		0.160		0.213		0.09	
	Age grade	F	11.47		14.29		3.22		0.09		1.10	
		p-value	0.001		< 0.001		0.075		0.761		0.295	
		*η*2	0.065		0.079		0.019		0.001		0.007	
	Gender	F	0.18		0.94		0.17		2.32		1.29	
	*∗*	p-value	0.669		0.332		0.675		0.129		0.257	
	Age grade	*η*2	0.001		0.006		0.001		0.014		0.008	

Adjusted for BMI z-score and wear time.

**Table 4:** Physical activity levels and sedentary time by gender and by age grade on weekends.

			**Sedentary time**		**Light activity**		**Moderate activity**		**Vigorous activity**		**Steps**	
			(min/day)		(min/day)		(min/day)		(min/day)		(steps/day)	
			Boys	Girls	Boys	Girls	Boys	Girls	Boys	Girls	Boys	Girls
**Children**	Mean		438.0	457.0	283.7	290.7	54.8	37.3	20.9	12.4	12265.0	9859.4
	95% CI	Lower	412.9	433.3	264.8	272.8	47.8	30.7	17.2	8.9	10921.4	8587.2
		Upper	463.0	480.7	302.6	308.5	61.8	43.9	24.7	16.1	13608.5	11131.6
**Adolescents**	Mean		481.7	508.3	247.1	243.4	46.4	34.9	22.3	10.8	11570.6	9333.4
	95% CI	Lower	457.6	483.7	228.9	224.8	39.7	28.1	18.7	7.1	10277.9	8012.5
		Upper	505.7	532.9	265.2	262.0	53.1	41.8	26.0	14.5	12863.4	10654.3
	Gender	F	3.45		0.03		17.8		28.9		12.3	
		p-value	0.065		0.861		< 0.001		< 0.001		0.001	
		*η*2	0.020		< 0.001		0.097		0.149		0.069	
	Age grade	F	14.7		20.14		2.46		0.003		0.84	
		p-value	< 0.001		< 0.001		0.119		0.957		0.360	
		*η*2	0.081		0.108		0.015		< 0.001		0.005	
	Gender	F	0.09		0.32		0.76		0.65		0.16	
	*∗*	p-value	0.760		0.571		0.382		0.420		0.899	
	Age grade	*η*2	0.001		0.002		0.005		0.004		< 0.001	

Adjusted for BMI z-score and wear time.

**Table 5:** Attainment of moderate to vigorous physical activity guideline by gender and by age grade.

		**B ± ES**	**OR**	**95% CI**		**p-value**
**Gender**	Boys	2.14 ± 0.35	8.569	4.239	17.231	< 0.001
	Girls	1				
**Age grade**	Children	-0.28 ± 0.31	0.750	0.409	1.378	0.354
	Adolescents	1				

## Data Availability

The data used to support the findings of this study is not freely available because the database is not entirely exploited. Access to these data will be considered by the author upon request.
